# Earplug Umbilicoplasty: A Simple Method to Prevent Umbilical Stenosis in a Tummy Tuck

**Published:** 2019-04-12

**Authors:** Swapnil Kachare, Christina Kapsalis, Milind Kachare, Andrea Hiller, Sara Abell, Thomas J. Lee, Bradon J. Wilhelmi

**Affiliations:** ^a^Division of Plastic and Reconstructive Surgery, Department of Surgery; ^b^School of Medicine, University of Louisville, Louisville, KY; ^c^Department of Surgery, Robert Wood Johnson Medical School, New Brunswick, NJ

**Keywords:** umbilicus, umbilicoplasty, abdominoplasty, stenosis, tummy tuck

## Abstract

**Objective:** An aesthetically pleasing umbilicus is a vital component of patient satisfaction following an abdominoplasty. An umbilicus that is moderate to small is desired to achieve the best aesthetic result, but a small umbilicus has potential for stenosis. This article presents a method for umbilical stenting that creates a modest umbilicus, while preventing stenosis. **Methods:** All patients underwent abdominoplasty with an umbilical reconstruction using an inverted U-flap method between 2015 and 2017. An earplug was placed into the umbilicus at 2 weeks postoperatively for a total of 4 to 6 week. Patients were evaluated subjectively on the aesthetic outcome. **Results:** Twenty-one female patients were evaluated 6 weeks postsurgery. In all cases, both the patient and the surgeon were 100% satisfied with the final size. Umbilical size ranged from 1.8 to 2.2 cm. **Conclusions:** Use of an earplug for umbilical stenting is a simple and reproducible method to create an aesthetically pleasing umbilicus and avoid stenosis.

Abdominoplasty has consistently remained one of the top cosmetic surgical procedures, with more than 127,500 procedures performed in 2016.^1^ During this procedure, the umbilicus is repositioned within the abdominal skin flap. Transposition of the umbilicus is also a component of procedures that involve autologous breast reconstruction using abdominal tissue, such as transverse rectus abdominis myocutaneous (TRAM) or deep inferior epigastric perforator (DIEP) flaps. Although umbilicoplasty may seem to be a minor component of these procedures, it is absolutely critical for obtaining the most cosmetically pleasing result. The final aesthetics of the umbilicus can ultimately influence patient satisfaction with the procedure, and a poor result can ruin an otherwise successful abdominoplasty.^2^

When glancing at an aesthetically pleasing female abdomen, the umbilicus is the central focus.^3^ It is a unique structure in that it is the only naturally occurring scar in the human body.^4^ The umbilicus is considered by many to be an aesthetic subunit in and of itself.^5^^,^^6^ When absent, misshapen, or displaced, it can create an unnatural looking abdomen that causes undue attention to the midsection, resulting in psychological distress for the patient.^7^ Position, size, shape, and depth of the umbilicus are characteristics that contribute to the overall aesthetics of the abdomen.^8^

Because of the negative aesthetic perception of a large and wide umbilicus, plastic surgeons attempt to create a modest umbilicus during umbilical reconstruction. However, the process of skin healing and scar remodeling varies from person to person, making complications unpredictable; thus, a relatively small umbilicus may become stenotic and distorted. Despite this commonly anticipated problem, there is no standardized approach to dealing with umbilical stenosis. We present a method of umbilical stenting that allows the surgeon to create a modest umbilicus while also avoiding the complication of umbilical stenosis.

## METHODS

**Figure 1:**
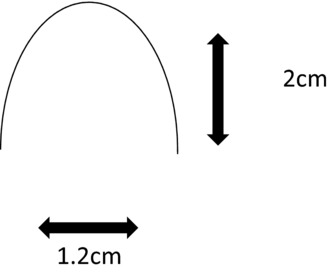
Inverted U-flap design.

Patients who underwent abdominoplasty between the years 2015 and 2017 comprised the study population. A single surgeon performed all operations with the assistance of plastic surgery residents. The inverted U-flap method was used for umbilical reconstruction (Fig 1). Postoperatively, patients were seen weekly, and on the second week, a moderately firm foam earplug was inserted into the umbilicus if the umbilicus incision had epithelialized and was nondraining. The plug was kept in place until 6 to 8 weeks postoperatively. A subjective assessment of aesthetic outcome was performed.

## RESULTS

**Figure 2:**
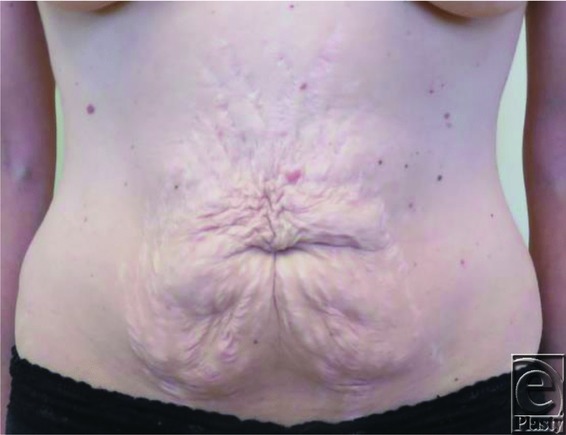
Preoperative umbilicus.

**Figure 3:**
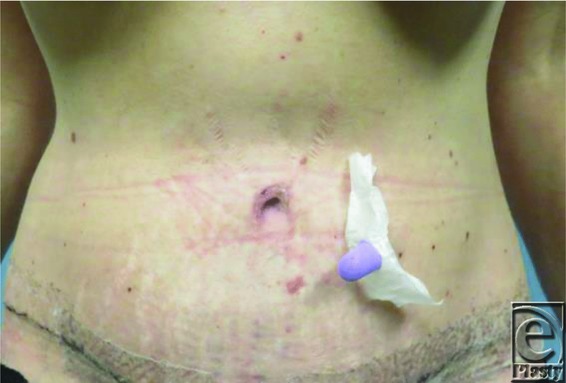
Placement of earplug at 2 weeks.

**Figure 4:**
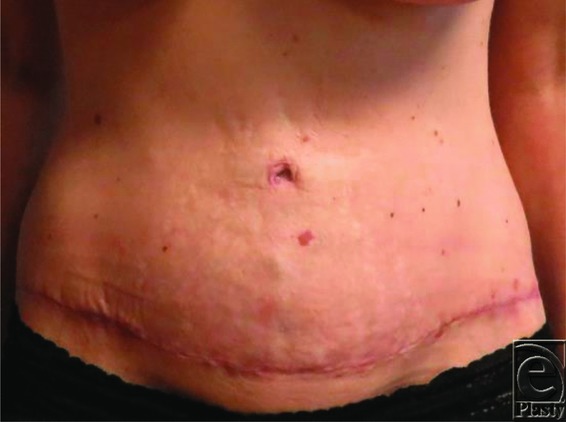
Poststenting.

A total of 21 patients comprised the study population. All patients were female, ranging in age from 42 to 63 years. Six weeks postoperatively, subjective assessments of the umbilicus were performed by both the surgeon and the patient (Figs 2-4). For each patient, both the surgeon and the patient had 100% satisfaction with the final size of the umbilicus, with no complaints of the umbilicus being too small or too large. The final size for the umbilicus ranged from 1.8 to 2.2 cm. There was no skin breakdown or ulcer formation secondary to the use of earplug.

## DISCUSSION

The umbilicus is a key component contributing to the overall aesthetics of the abdomen.^9^ It draws attention to the curve of the inferior abdomen as it helps define the medial abdominal sulcus. An aesthetically appealing umbilicus is one that is relatively small, vertically oriented, and oval-shaped.^8^^-^11 However, if the umbilicus is large, misplaced, or distorted, it may draw negative attention to the central abdomen.^9^ In this study, we describe a simple and reproducible method to create a small, nonstenotic, and attractive umbilicus.

Stenosis is a known complication following umbilical reconstruction, either as an isolated procedure or as part of an abdominoplasty or breast reconstruction procedure. Surgical techniques such as scar revisions and placements of stents have been described as potential options for prevention and management of umbilical stenosis^7^^,^^12^^,^^13^; however, no standardized method exists (Table 1). Similar to our group, Dini and Ferreira^13^ first proposed the use of a simple earplug to combat umbilical stenosis. In contrast, they used softer earplugs, while also recommending the use of a firmer consistency earplug as it would provide a stronger power of dilation. The time and duration of placement in relation to the umbilicoplasty were not provided.^13^ Barbosa et al^14^ advised the use of a vaginal tampon immediately following reconstruction that was replaced when wet, with transition to silicone plugs or soft earplugs at 15 days; however, duration of plug placement was not provided. Pons and colleagues^15^ described the use of a marble to manage stenosis, stating that its hardness exceed that of other stenotic devices. Similar to our group, they placed the stent 3 weeks postoperatively for a total of 2 months. However, unlike an earplug, adhesive was required to hold the marble in place.^15^ We placed a firm foam plug at 2 weeks postoperatively, for a total of 4 to 6 weeks, with no issues related to wound healing, skin problems, necrosis, infection, stenosis, or enlarged umbilicus.

Many have explored the components of an aesthetically appealing umbilicus^9^^,^^8^^,^^10^^,^^11^^,^^16^ but few have proposed solutions to combat the umbilical stenosis that occurs postoperatively.^12^^,^^13^^,^^15^ As shown in Table 2, summarized from an article by Joseph et al,^10^ many incision types have been described for the ideal umbilicus. A surgical technique described by Baack et al^12^ involves using a double-opposing Z-plasty designed around a curve, rather than the traditional straight scar. By introducing a curve, the superior umbilical hood can be created while the stenosis is relieved, and all the suture lines are placed in the umbilicus. The downside of this technique is that it is more invasive than other nonsurgical options.

Characteristics of the aesthetically appealing umbilicus include a relatively small, vertically oriented, and oval-shaped umbilicus with slight superior hooding or no hooding at all.^8^^-^11 The presence of a large and wide umbilicus that is transversely oriented is considered unattractive and is associated with aging and weight gain.^9^^,^^11^ Therefore, one goal following an abdominoplasty is to avoid making the umbilicus too big, as making the umbilicus too big may be much harder to correct than making an umbilicus too small. In our study, an inverted U technique allowed for a natural appearing umbilicus, with the use of an earplug to prevent stenosis, thereby creating an aesthetically pleasing umbilicus. Utilizing the inferiorly based flap with the inverted U technique avoids the complications associated with a circular scar, such as contracture, which would result in a small stenotic umbilicus. In addition, it allows for the re-creation of superior hooding, which is able to hide a portion of the scar, leading to a natural, youthful umbilicus that has been demonstrated to have an increased level of patient satisfaction.^17^

Our study is limited by a small sample size and being a single-institution study; however, every patient had a consistently appropriate-sized umbilicus that was not too small or too large. Use of a superior hood method for umbilicoplasty and placement of an earplug allows for a consistent umbilicus size, without stenosis.

## Figures and Tables

**Table 1:** Stenting methods

Authors	Year	Stent
Dini and Ferreira^13^	2006	Soft earplug
Barbosa et al^14^	2008	Tampon
Pons et al^15^	2013	Marble

**Table 2:** Umbilicoplasty techniques^10^

Authors	Year	Incision type
Malic et al^17^	2007	Round
Malic et al^17^	2007	Inverted U
Castillo et al^18^	2007	Y-shaped
Rozen et al^19^	2007	Vertical oval
Bruekers et al^20^	2009	Vertical ellipse
Dogan^21^	2010	Vertical line
Pallua et al^11^	2010	Inverted V
Mazzocchi et al^22^	2011	Double-opposing Y
Lesavoy et al^23^	2012	Inverted V chevron
Rodriguez-Feliz et al^24^	2012	Vertical ellipse
